# Developing an Allogeneic Hematopoietic Progenitor Cell Transplant Service in a Resource-Limited Country: Challenges and Outcomes

**DOI:** 10.7759/cureus.44818

**Published:** 2023-09-07

**Authors:** Uzma R Mahar, Bushra Ahsan, Usman Ahmad, Syed W Bokhari

**Affiliations:** 1 Medical Oncology, Shaukat Khanum Memorial Cancer Hospital and Research Centre, Lahore, PAK

**Keywords:** resource-limited country, acute myeloid leukemia (aml), acute lymphoblastic leukemia (all), outcomes of allograft., allogenic stem cell transplant

## Abstract

Introduction

Allogeneic stem cell transplant has curative potential for many hematological disorders. Building an allogeneic hematopoietic progenitor cell transplant (HPCT) unit requires huge investment, infrastructure, equipment, medical supplies, and training of health care professionals. The key objective of this study is to share our experience of developing an allogeneic HPCT service at our tertiary care cancer hospital in a low-middle-income country. In addition, this study presents the outcomes of the first 30 allogeneic HPCTs done at our center.

Methods

This retrospective observational study included adult patients 18 years old or older with hematological malignancies who underwent allogeneic HPCT between July 2019 and April 2023 at Shaukat Khanum Memorial Cancer Hospital and Research Centre.

Result

Of the 30 patients, 24 underwent matched sibling donor (MSD) transplants in which a myeloablative-conditioning regimen (MAC) was used in 19, and a reduced conditioning regimen (RIC) was used in one. Of the six haploidentical-related donor transplants, four received MAC, and two received RIC. The median recipient age at HPCT was 23 and 21 years for MSD and Haplo-related donor transplants, respectively. The median follow-up duration was 12 months (Range: 10 days - 33 months). The overall survival rate at one year was 71.3% among all allogeneic stem cell transplant patients, whereas the disease-free survival rate at one year was 63.7%. In the acute lymphoblastic leukemia group, the disease-free survival rate at one year post allograft was 51.5%, while in the acute myeloid leukemia group, it was 78.7%.

Conclusion

This study demonstrates the successful development of an allogeneic bone marrow transplant unit at our hospital despite significant financial constraints. This has allowed us to provide a potentially curative and life-saving treatment to a substantial number of cancer patients. The bone marrow transplant outcomes of this study are comparable to those reported by international bone marrow transplant registries.

## Introduction

Allogeneic hematopoietic progenitor cell transplant (HPCT) has curative potential for many malignant and non-malignant hematological disorders. HPCT has become the standard of care for many disorders in developed countries; however, its complexity and cost have made it difficult for wide accessibility in developing countries. Building an allogeneic HPCT unit requires huge investment, infrastructure, equipment, medical supplies, and training of health care professionals [1,2]

The strategy of setting up an allogeneic HPCT unit varies among different centers depending on the available structure, finances, case variety, and load. Fundamental components of a hematopoietic stem cell transplant center include blood banking, apheresis capacity, stem cell processing, and storage, which requires specialized equipment, trained staff, and standard operating protocols [1]. The availability of an advanced diagnostic laboratory with facilities to perform human leukocyte antigen (HLA) typing, flow cytometry, cytogenetics, cytology, fluorescence in situ hybridization (FISH), and chimeric studies is essential and can reduce the challenges and overall cost of transplants if performed in-house or outsourced to neighboring transplant centers depending on the workload [3].

Another important aspect of establishing a transplant unit is to ensure measures to reduce the risk of infection, such as adherence of healthcare providers to meticulous infection prevention practices, including hand hygiene and the proper use of personal protective equipment [4]. Moreover, educating patients and their visitors about infection prevention measures empowers them to actively participate in their safety, which enables the prevention of infection during the transplant process. In developing regions of the world, poor environmental conditions and higher levels of antimicrobial resistance make it challenging and crucial to design a transplant unit with strict environmental controls. In this regard, international guidelines advocate using high-efficiency particulate air filtration and positive-pressure bone marrow transplant rooms [5].

Bone marrow transplantation requires multidisciplinary team support, including radiation oncology for total body irradiation required for conditioning and anesthesia for apheresis/central line insertion used for stem cell collection, conditioning, and infusion. Several different specialty consultants, such as infectious disease, cardiology, nephrology, nutrition, psychiatry, and intensive care, are also needed per the international accreditation guidelines [6]. Furthermore, strong pharmaceutical support is pivotal for provisional drugs used specifically in allogeneic HPCT [7].

The key objective of this study is to share our experience developing an allogeneic HPCT service at our tertiary care cancer hospital in a low-middle-income country. In addition, this study presents the outcomes of the first 30 allogeneic HPCTs done at our center.

## Materials and methods

Study design and setting

This was an observational, retrospective study done at the Shaukat Khanum Memorial Cancer Hospital and Research Center, Lahore.

Inclusion criteria

All patients 18 years old or older with hematological malignancies who underwent allogeneic stem cell transplants between July 2019 and April 2023 at Shaukat Khanum Memorial Cancer Hospital and Research Centre were included in the present study. Allogenic transplant indications included were high-risk acute lymphoblastic leukemia or acute myeloid leukemia in first remission; relapsed acute lymphoblastic leukemia or acute myeloid leukemia in second complete remission; chronic myeloid leukemia either blast crisis in first remission or refractory to at least two tyrosine kinase inhibitors; and relapsed Hodgkin lymphoma after autologous stem cell transplant.

Exclusion criteria

Patients under 18 years of age or those who had benign hematological disease or active solid organ malignancy were not included. Patients who were unable to understand the process of transplant and follow-up were considered ineligible for transplant and hence excluded.

Data collection

Data, including age, sex, diagnosis, disease status at the time of transplant, and transplant-related complications, such as graft versus host disease, cytomegalovirus (CMV) reactivation, and graft failure, were collected from the hospital information system.

Statistical analysis

Data on all important outcomes post-HPCT, including infections, engraftment, non-relapsed mortality, relapse, progression-free survival, and overall survival, were analyzed using GraphPad Prism 9.5.1 software.

## Results

Of the 30 patients, 24 patients underwent matched sibling donor (MSD) transplants using a myeloablative-conditioning regimen (MAC) for 19 and a reduced conditioning regimen (RIC) for one patient. Of the six haplo-related donor transplants, three received MAC, and two received RIC. The median recipient age at HPCT was 23 and 21 years for MSD and haplo-related donor transplants, respectively. Fifteen patients in the MSD group and five in the haplo-related donor group were gender-matched with the donor; the rest were gender mismatched (Table 1). All transplant-eligible patients and donors underwent a thorough pre-transplant (recipient and donor) assessment that included an examination of the patient's performance status; baseline blood, urine, and stool tests; organ function assessment; and viral serology, etc. Hematopoietic Cell Transplantation Comorbidity Index (HPCT-CI) was calculated for each recipient, and after discussion in the bone marrow transplant (BMT) multidisciplinary team meeting, the recipient and donor eligibility and intensity of the conditioning regimen and protocol were finalized. Donor-specific antibody screening was done for haplo-related donor HPCT recipients however none of the recipients was positive for donor-specific antibody.

**Table 1:** Recipient and donor characteristics MSD - matched sibling donor; HPCT - hematopoietic progenitor cell transplant; haplo - haploidentical; AML - acute myeloid leukemia; ALL - acute lymphoblastic leukemia; CML - chronic myeloid leukemia; MAC - myeloablative conditioning; RIC - reduced intensity conditioning; Cy - cyclophosphamide; FLU - fludarabine; TBI - total body irradiation; MEL - melphalan; MTX - methotrexate; PTCy - post-transplant cyclophosphamide; CMV - cytomegalovirus; HPC - hematopoietic progenitor cell; GVHD - graft versus host disease

Characteristics	MSD-HPCT (n=24)	Haplo-related donor HPCT (n=6)
Age (year)	Mean, median (range)	Mean, median (range)
Recipient	23.5, 23 (18-36)	20.8, 21 (18-25)
Donor	26.1, 25 (16-40)	35.6, 30.5 (20-56)
Sex (recipient/donor)	n (%)	n (%)
Male/female	8 (33.3)	1 (16.6)
Female/female	3 (12.5)	1 (16.6)
Male/male	12 (50)	4 (66.6)
Female/male	1 (4.1)	-
Donor relation	n (%)	n (%)
Sibling	24 (100)	4 (60)
Parent	0 (0)	2 (40)
Disease	n (%)	n (%)
AML	9 (37.5)	3 (50)
ALL	11 (45.8)	3 (50)
CML	3 (12.5)	-
Classic Hodgkin lymphoma	1 (4.1)	-
Conditioning intensity	n (%)	n (%)
MAC	22 (91.6)	4 (66.66)
RIC	2 (8.3)	2 (33.3)
MAC regimen	n (%)	n (%)
Cy/TBI	22 (91.6)	-
Flu/TBI	-	4 (66.66)
RIC regimen	n (%)	n (%)
MEL/FLU/TBI	-	2 (33.3)
FLU/MEL	2 (8.3)	-
GVHD prophylaxis	Cyclosporine + MTX	PTCy + Tacrolimus + MMF (mycophenolate mofetil)
CMV serostatus (recipient/donor)	n (%)	n (%)
Positive/positive	23 (95.8)	6 (100)
Positive/negative	1 (4.1)	-
ABO status	n (%)	n (%)
ABO matched	19 (79.16)	3 (50)
Minor mismatch	2 (8.33)	1 (16.66)
Major mismatch	2 (8.33)	2 (33.33)
Bidirectional mismatch	1 (4.16)	-
HPC dose (CD 34 + cells)	2.75-10 million cells/kg	3-8.4 million cells/kg

Engraftment and graft failure

The median time to neutrophil engraftment was 16 and 13 days for the MSD and haplo-related donor transplants, respectively (Table 2). No significant differences were noted in neutrophil or platelet engraftment with different intensities of conditioning regimens. The median time to platelet engraftment was 17.5 days for MSD-HPCT and 17 days for haplo-related donor HPCT (Table 2). One patient who underwent haplo-related donor HSCT had primary graft failure, whereas none of the HPCT patients reported secondary graft failure.

**Table 2:** Neutrophil and platelet engraftment MSD - matched sibling donor; Haplo - haploidentical; HPCT - hematopoietic progenitor cell transplant; MAC - myeloablative conditioning; RIC - reduced intensity conditioning

Parameter	MSD-HPCT	Haplo-related donor HPCT
Neutrophil engraftment, median day (range)		
All patients	16 (12-20)	13 (11-18)
MAC	16 (12-20)	12 (11-15)
RIC	12 (None)	16 (14-18)
Platelet engraftment, median day (range)		
All patients	17.5 (12-21)	17 (12-32)
MAC	18 (12-21)	12.5 (12-21)
RIC	13 (None)	28 (24-32)

Graft versus host disease

The cumulative incidence of grade II-IV acute graft versus host disease (aGVHD) was 25.9%; in matched sibling donor HPCTs, it was 23.8%, and in haplo-related donor HPCTs, it was 33.3% (Table 3). The median time to development of aGVHD was 41.5 days (Range: 28-73 days). Of these, 14.2% and 33.3% were steroid-refractory aGVHD (SR aGVHD) in the MSD-HPCTs and haplo-related donor HPCTs, respectively. Those who had SR aGVHD were treated with a second-line therapy such as ruxolitinb (n=5), etanercept (n=1), and sirolimus (n=1).

**Table 3:** Complications of allogeneic stem cell transplant MSD - matched sibling donor; Haplo - haploidentical; HPCT - hematopoietic progenitor cell transplant; aGVHD - acute graft versus host disease; SR - steroid refractory; cGVHD - chronic graft versus host disease; CMV - cytomegalovirus; VOD/SOS - veno-occlusive disease/sinusoidal obstruction syndrome

Complications	MSD-HPCT (n=24)	Haplo-related donor HPCT (n=6)	Overall (n=30)
Infection during HPCT (within 30 days)	83.3% (n=20/24)	83.3% (n=5/6)	83.3% (n=25/30)
aGVHD grade II-IV	23.8% (n=5/21)	33.3% (n=2/6)	25.9% (n=7/27)
aGVHD grade III-IV	14.2% (n=3/21)	33.3% (n=2/6)	18.5% (n=5/27)
SR aGVHD	14.2% (n=3/21)	33.3% (n=2/6)	18.5% (n=5/27)
Therapy requiring cGVHD	31.5% (n=6/19)	33.3% (n=2/6)	32% (n=8/25)
CMV reactivation	70.8% (n=17/24)	100% (n=6/6)	76.6% (n=23/30)
Hemorrhagic cystitis	16.6% (n=4/24)	33.3% (n=2/6)	20% (n=6/30)
VOD/SOS	4.1% (n=1/24)	16.6% (n=1/6)	6.6% (n=2/30)
Primary graft failure	0% (n=0/24)	16.6% (n=1/6)	3.3% (n=1/30)
Transplant-related mortality (day 30)	12.5% (n=3/24)	16.6% (n=1/6)	13.3% (n=4/30)

The overall incidence of chronic graft versus host disease (cGVHD) that required therapy was 32%. Around 31.5% of patients who underwent MSD-HPCTs and 33.3% with haplo-related donor HPCTs required therapy for cGVHD (Table 3). The median time to develop therapy-requiring cGVHD was 194 days (Range: 127-377 days). Three patients had limited skin cGVHD. Among the cGVHD requiring therapy oral cavity was the most common organ involved (n=7), followed by skin (n=6), liver (n=6), GI tract (n=2), and joints (n=1). Three patients had steroid refractory cGVHD.

Infections during HPCT

Despite antimicrobial prophylaxis, around 83.3% of the patients had infections during HPCTs (Table 3). At our center, antibacterial (quinolone) and anti-fungal (voriconazole) prophylaxis are started from day -7 and are continued until discharge and day +90, respectively. Anti-pneumocystis pneumonia (PCP) prophylaxis commenced on day -7 and stopped on day -1 before the engraftment and then was restarted to one year post allograft. According to our hospital antibiogram of 2020, the most isolated gram-negative organism was *Escherichia coli*, and around 23% of the strains showed resistance to one of the three carbapenems. Among the gram-positive organisms, *Staphylococcus aureus* was the most prevalent, with 50% being methicillin-resistant strains. The most common cause of transplant-related mortality in our HPCT patients was sepsis (n=4/5), and of these, two had carbapenem-resistant organisms in blood.

CMV reactivation

All recipients and donors were seropositive for CMV before the transplant, except for one donor. CMV polymerase chain reaction (PCR) monitoring was started one week after stem cell infusion until day +90 post allograft. Patients received high-dose acyclovir for anti-CMV prophylaxis due to the unavailability and high cost of letermovir. Around 70.8% of the MSD-HPCT recipients and 100% of the haplo-related HPCT recipients had CMV reactivation (Table 3). The majority of the CMV reactivation cases were treated with ganciclovir and valganciclovir, while foscarnet was used exclusively to treat patients experiencing cytopenias. There was only one case of refractory CMV infection, and the patient did not survive. The overall CMV reactivation was noted in 76.6% of the allo-HPCTs (Table 3).

Hemorrhagic cystitis

Of the 30 patients, six developed late-onset hemorrhagic cystitis (HC). None of the patients suffered from early-onset HC. The incidence of HC was double in haplo-related donor HPCTs (33.3%) and was 16.6% in MSD-HPCTs, probably due to the use of post-transplant cyclophosphamide (PTCy) as a graft versus host disease (GVHD) prophylaxis in haplo-HPCTs (Table 3). All patients were found to have BK virus in their urine and grade III-IV HC. These patients were managed with IV fluids, diuretics, oral estrogen, and bladder irrigation. Two patients required cystoscopy and cauterization of bleeders. The median number of days from allograft to HC was 26 days (range 15-103 days).

Relapse and transplant-related mortality

For the whole cohort, the rates of transplant-related mortality (TRM) at day 30 and day 100 were 13.3% and 16.6% (n=5/30), respectively (Table 3). Day 30 and day 100 TRM in the matched sibling donor transplants was 12.5%, whereas, in the haplo-related donor transplants, day 30 and day 100 TRM was 16.6%. The most common cause of TRM was sepsis (n=4/5). Two patients died of diffuse alveolar hemorrhage; among them, one had cyclophosphamide-induced cardiac toxicity and died from multi-organ failure. One patient had refractory CMV reactivation followed by graft failure, and another had severe aGVHD, BK virus HC, and eventually passed away due to sepsis.

Five out of the twenty-four patients who underwent matched sibling donor transplants relapsed. Four of the relapsed patients had acute lymphoblastic leukemia (n=4/11), and one had chronic myeloid leukemia (n: 1/3). Those patients who relapsed received a MAC regimen. The median time of relapse post allograft was 303 days (range 57-641 days). No patient in the haplo-related donor transplant group relapsed. The relapse rate among all patients was 16.6%. 

Survival

**Figure 1:**
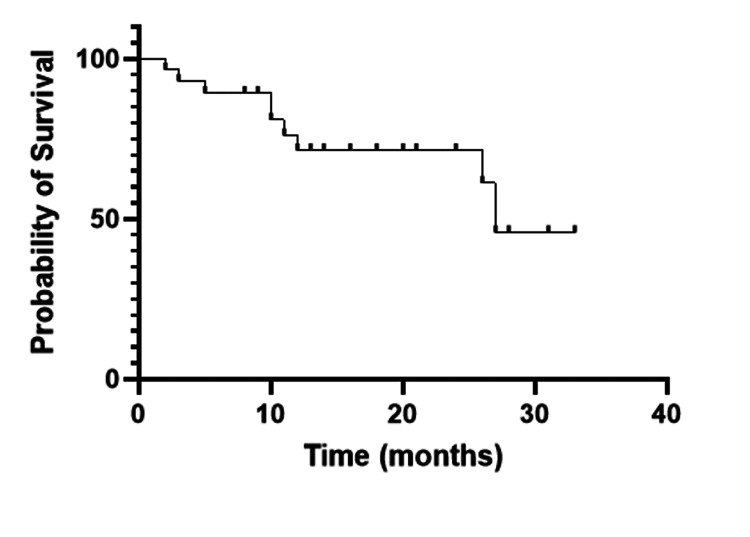
One-year overall survival of the whole cohort

**Figure 2:**
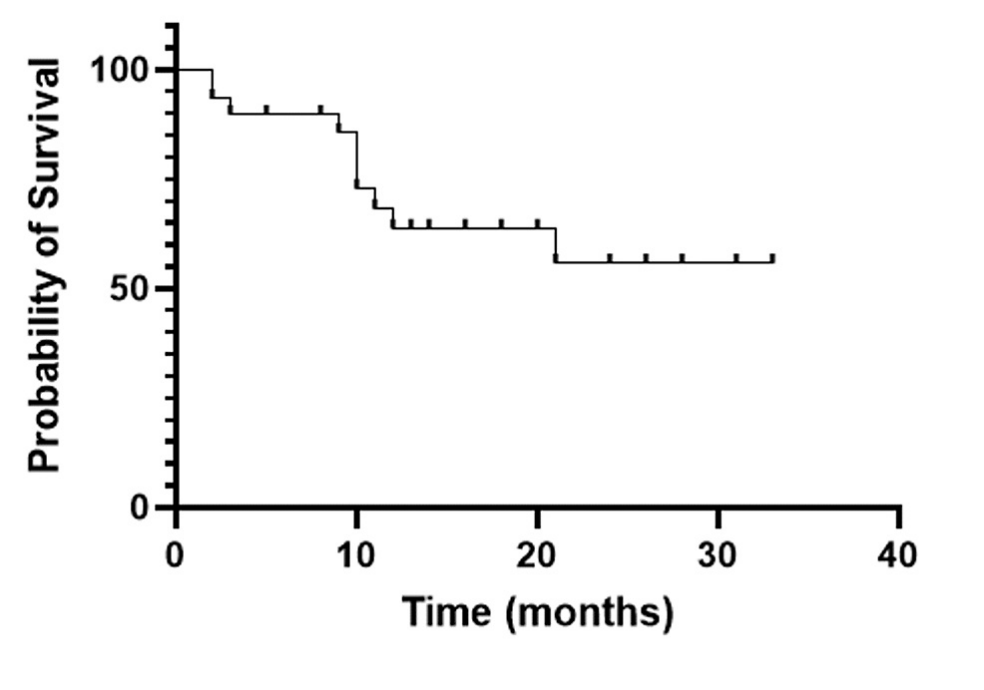
One-year disease-free survival of the whole cohort

**Figure 3:**
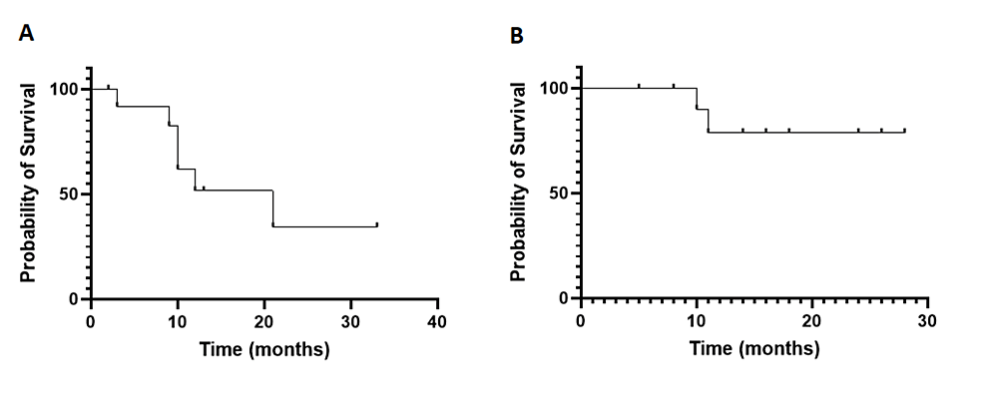
One-year disease-free survival in acute lymphoblastic leukemia group (A) and acute myeloid leukemia group (B)

The overall survival rate at one year was 71.3% among all allogeneic stem cell transplant patients (Figure 1), whereas the disease-free survival rate at one year was 63.7% (Figure 2). The median follow-up duration was 12 months (range 10 days - 33 months). In the acute lymphoblastic leukemia group, the disease-free survival rate at one year post allograft was 51.5%, while in the acute myeloid leukemia group, it was 78.7%, respectively (Figure 3).

## Discussion

Shaukat Khanum Memorial Cancer Hospital and Research Center is located in Lahore and is the only Joint Commission International (JCI) accredited welfare hospital that provides free-of-cost, state-of-the-art facilities to cancer patients. The hospital serves not only the population of Lahore, which is around 13.13 million, but also caters to cancer patients from various parts of Punjab, the largest province in Pakistan. Allogeneic stem cell transplants for adults were a much-needed service at our center and were started in July 2019 and aimed to rescue patients with blood cancers. Our hospital has always prioritized the collaborative efforts of multidisciplinary teams in delivering exceptional healthcare. Recognizing the critical need for expertise in bone marrow transplants, we took decisive action by collecting funds and facilitating the training of dedicated healthcare staff at a neighboring bone marrow transplant center and later with in-house training and workshops. 

Despite limited financial resources, we remained committed to providing optimal care and established a specialized four-bed unit dedicated to allografts. To ensure the highest level of safety and infection control, we installed a centralized high-efficiency particulate air (HEPA) filter attached to the unit, as recommended by FACT-JACIE [8]. HEPA filters, renowned for their exceptional filtration capabilities, efficiently capture and eliminate airborne pathogens and contaminants, decreasing the risk of fatal infections, such as invasive aspergillosis, and spreading infections within the facility [9,10]. This investment underscored our commitment to providing the best possible care to allo-HPCT patients, even in the face of financial constraints. This proactive approach, along with antibiotic stewardship, was vital in combating the increasing antibiotic resistance found particularly in low and middle-income countries, where high infection rates and the prevalence of antibiotic-resistant bacteria pose significant challenges [11].

HLA typing is a mandatory requirement of HPCT. The gold standard for HLA typing is high resolution, which detects HLA mismatches at the allelic level using advanced molecular techniques such as next-generation sequencing (NGS) and PCR [12,13]. However, achieving greater depth in HLA resolution results in higher costs. Due to financial limitations at our center, we currently perform low-resolution HLA typing using a sequence-specific oligonucleotide probing method [14]. We do not have a donor registry available in our country, so all the allografts carried out were either fully matched sibling donor transplants or haplo-identical related donor transplants.

The molecular laboratory was further upgraded to conduct chimerism analysis using the DNA fragment analysis technique known as short tandem repeats (STR). This allows us to detect loss of chimerism and predict relapse, helping us navigate the duration of immunosuppressants. Another technique utilized for chimeric studies is NGS, which is more expensive and sensitive [15]. While lineage-specific chimerism is more sensitive in early relapse prediction during HPCT, we currently perform chimerism analysis on whole blood due to the high cost and expertise required for lineage-specific testing [16].

The autologous stem cell transplantation facility was already functioning at our center, so providing irradiated blood products to HPCT recipients was available using gamma radiation, as recommended [17]. We equipped our hospital with a stem cell-processing laboratory within the blood bank. Following stem cell collection in the blood bank using two different apheresis machines, Fresenius and Spectra Optia, we extract the CD34 cell enumeration sample in the laboratory with precision and send it for analysis using a single platform method, flow cytometry technique to ensure an accurate assessment of the stem cell dose [18].

In addition, a cryopreservation facility was established within the blood bank by utilizing dimethyl sulfoxide (DMSO) as the cryoprotectant. Stem cells are safely stored for one year at an ultra-low temperature of -80°C within meticulously maintained mechanical refrigerators [19,20]. While liquid nitrogen refrigerators are well known for their effectiveness in cryopreservation and recommended by the FACT-JACIE, we opted for mechanical refrigerators due to their lower cost and more manageable maintenance requirements [21]. Although we rarely require cryopreservation in the allogeneic HPCT setting, we have a facility with backup mechanical freezers if required. Our cryopreservation techniques ensure the durable viability and availability of stem cells, reflected by the timely engraftment of our patients after stem cell/graft infusions.

Currently, we have a bone marrow transplant team comprised of three BMT physicians, including the BMT lead, ten BMT nurses, and two coordinators. Regular weekly multidisciplinary team meetings and monthly meetings with the pharmacy are conducted to ensure the availability of bone marrow transplant-specific medicines. We organize quarterly quality management meetings to ensure the provision of quality care best to our capacity to transplant recipients and donors. The BMT evaluation committee members, which include higher hospital management, review and approve each allograft case to ensure the availability of finances before heading for the allogeneic stem cell transplant. From 2020 onwards, our BMT center has attained its Center for International Blood and Marrow Transplant Research (CIBMTR) membership, and we routinely submit data to the international registry for BMT-specific research. 

The limitation of the present study is that our center is performing allografts for hematological malignancies only. Moreover, this study is retrospective with a small sample size, and the median follow-up was only one year, so the strength of the present study is limited.

## Conclusions

This study demonstrates the accomplishments of an allogeneic bone marrow transplant unit at our hospital with the delivery of comparable transplant outcomes despite significant financial constraints. This achievement has allowed us to provide this potential curative and life-saving treatment to a substantial number of cancer patients. Our transplant outcomes are comparable to those reported by international bone marrow transplant registries. This study may encourage, help, and guide other centers in low-middle-income countries in developing and delivering allogeneic transplant services.
